# Supplementary material for: The Metabolic Profile of Anchusa officinalis L. Differs According to Its Associated Arbuscular Mycorrhizal Fungi
Source: Metabolites. 2022 Jun 22;12(7):573. doi: 10.3390/metabo12070573 (PMC9319164; doi:10.3390/metabo12070573)
Supplement: Supplementary file 1 [file metabolites-12-00573-s001.zip › metabolites-1745047-supplementary.pdf]

Supplementary materials

The metabolic profile of *Anchusa officinalis* L. differs according to its associated arbuscular mycorrhizal fungi

Evangelia Tsiokanos [1†], Annalisa Cartabia [2†], Nikolaos Tsafantakis [1], Ismahen Lalaymia [2], Aikaterini Termentzi [3], Maria Miguel [4], Stéphane Declerck [2], Nikolas Fokialakis [1*]

1 Department of Pharmacy, Division of Pharmacognosy and Natural Product Chemistry, National and Kapodistrian University of Athens, 15771 Athens, Greece; evatsiokanos@pharm.uoa.gr (E.T.); ntsafantakis@pharm.uoa.gr (N.T.), fokialakis@pharm.uoa.gr (N.F.)

2 Applied Microbiology, Mycology, Earth and Life Institute, Université catholique de Louvain-la-Neuve, 1348 Belgium; annalisa.cartabia@uclouvain.be (A.C.); ismahen.lalaymia@uclouvain.be (I.L.); stephan.declerck@uclouvain.be (S.D.)

3 Laboratory of Pesticides' Toxicolology, Benaki Phytopathological Institute, 8 St. Delta Street Kifissia, 14561 Athens, Greece; a.termentzi@bpi.gr

4 Instituto de Tecnologia Química e Biológica António Xavier, Universidade Nova de Lisboa (ITQB NOVA), Oeiras, Portugal; pmariamiguel@gmail.com (M.M.)

* Correspondence: fokialakis@pharm.uoa.gr; Tel.: +30-2107274524

† These authors contributed equally to this work.

Supplementary Figure S1. Principal component analysis (PCA) – Comparison of UHPLC-HRMS metabolic profiles from *A. officinalis* root (**A**) and shoot (**B**) samples associated with four different AMF species after 9 days of growth in the semi-hydroponic cultivation system (*R. irregularis* MUCL 41833: blue dots; *R. intraradices* MUCL 49410: green dots; *R. clarus* MUCL 46238-M[clarus]: red dots).

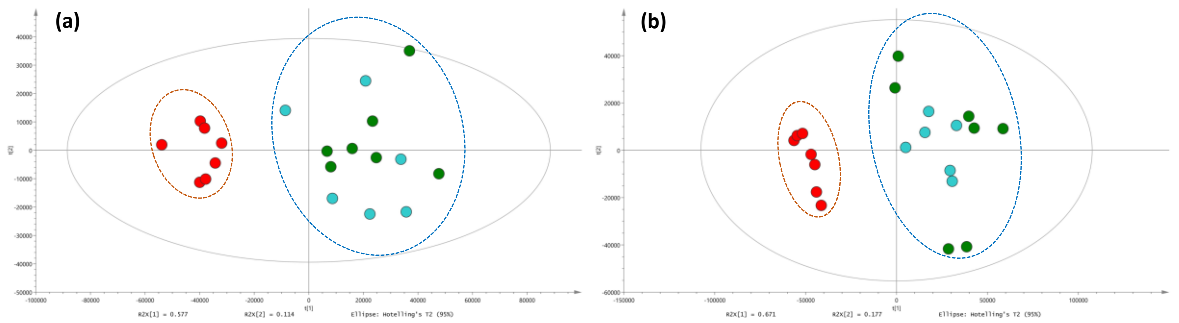

Supplementary Figure S2. Principal component analysis (PCA) – Comparison of UHPLC-HRMS metabolic profiles from *A. officinalis* root (**a**) and shoot (**b**) samples associated with four different AMF species after 9 days of growth in the semi-hydroponic cultivation system (*R. irregularis* MUCL 41833: blue dots; *R. intraradices* MUCL 49410: green dots; *R. aggregatus* MUCL 49408: yellow dots).

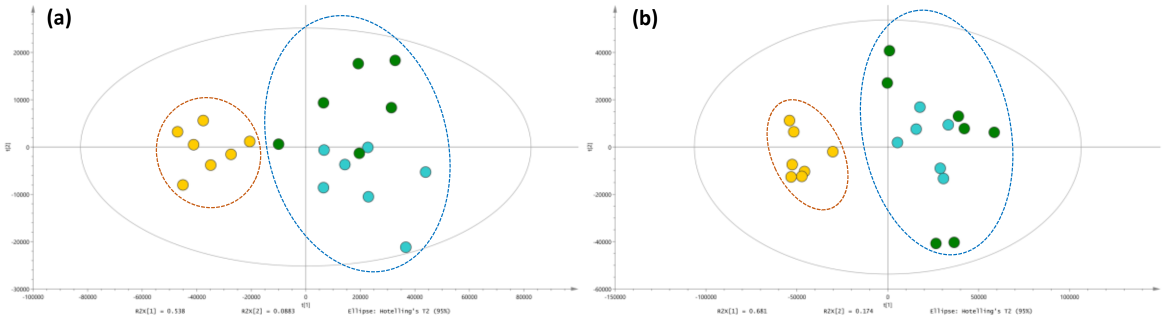

Supplementary Figure S3. Principal component analysis (PCA) – Comparison of UHPLC-HRMS metabolic profiles from *A. officinalis* root (**a**) and shoot (**b**) samples associated with four different AMF species after 9 days of growth in the semi-hydroponic cultivation system (*R. intraradices* MUCL 49410-M^{intra}: green dots; *R. clarus* MUCL 46238-M^{clarus}: red dots; *R. aggregatus* MUCL 49408-M^{aggreg}: yellow dots).

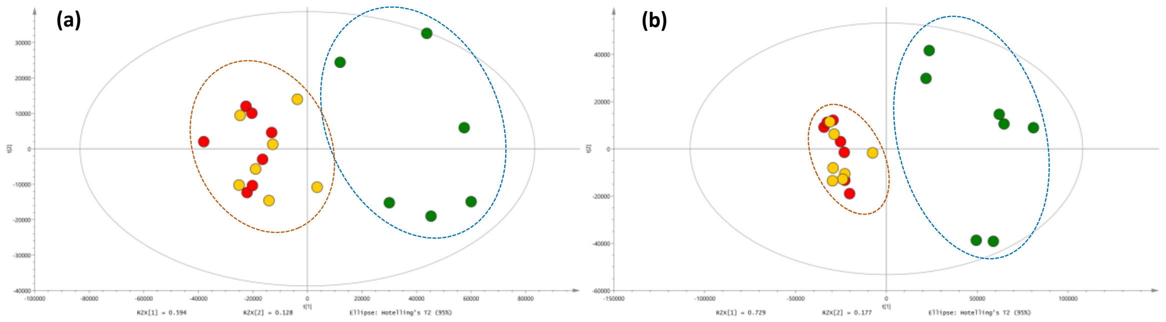

Supplementary Figure S4. Partial least square analysis – Discriminant analysis (PLS-DA) and permutation test (100 rearrangements) – Comparison of UHPLC-HRMS metabolic profiles from *A. officinalis* root (**a**) and shoot (**b**) samples associated with four different AMF species after 9 days of growth in the S-H cultivation system (*R. irregularis* MUCL 41833: blue dots; *R. intraradices* MUCL 49410: green dots).

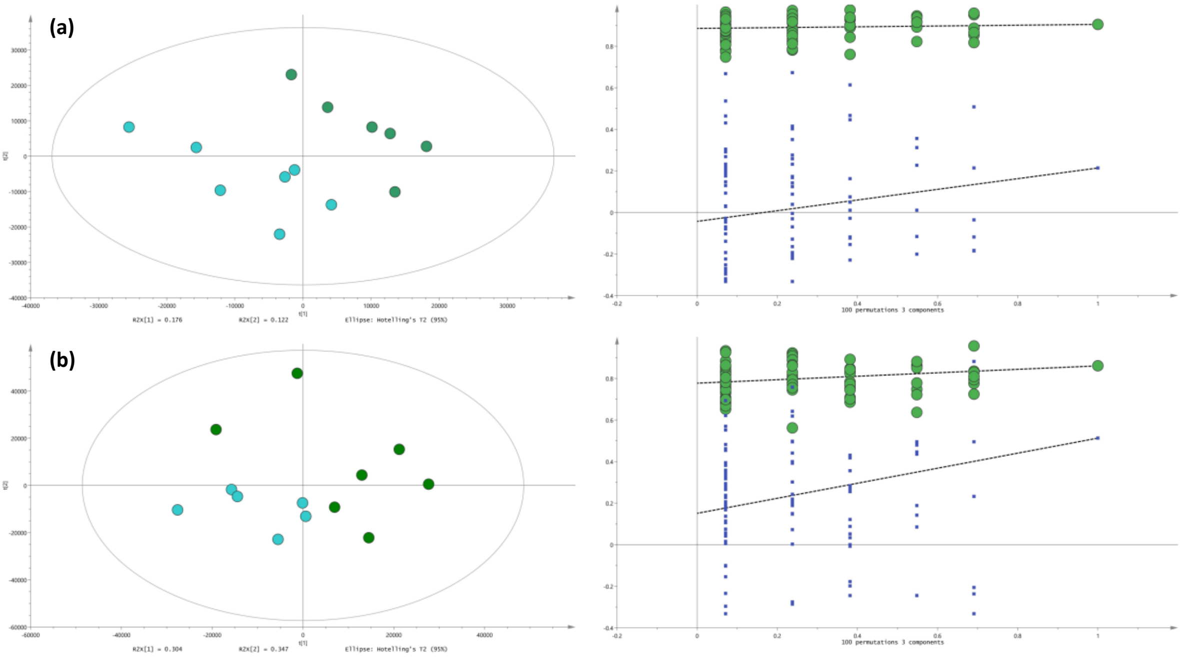

Supplementary Figure S5. Partial least square analysis – Discriminant analysis (PLS-DA) and permutation test (100 rearrangements) – Comparison of UHPLC-HRMS metabolic profiles from *A. officinalis* root (**a**) and shoot (**b**) samples associated with four different AMF species after 9 days of growth in the semi-hydroponic cultivation system (*R. clarus* MUCL 46238: red dots; *R. aggregatus* MUCL 49408: yellow dots).

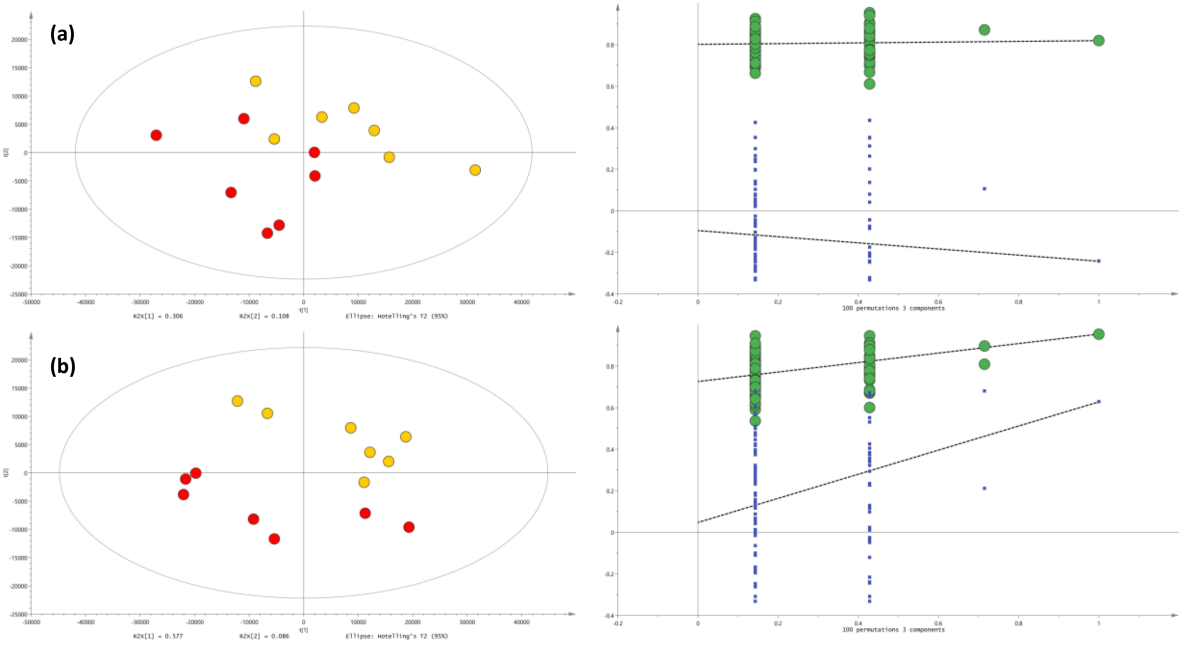

Supplementary Figure S6. Partial least square analysis – Discriminant analysis (PLS-DA) and permutation test (100 rearrangements) – Comparison of UHPLC-HRMS metabolic profiles from *A. officinalis* root (**a**) and shoot (**b**) samples associated with four different AMF species after 9 days of growth in the semi-hydroponic cultivation system (*R. irregularis* MUCL 41833: blue dots; *R. aggregatus* MUCL 46238: yellow dots).

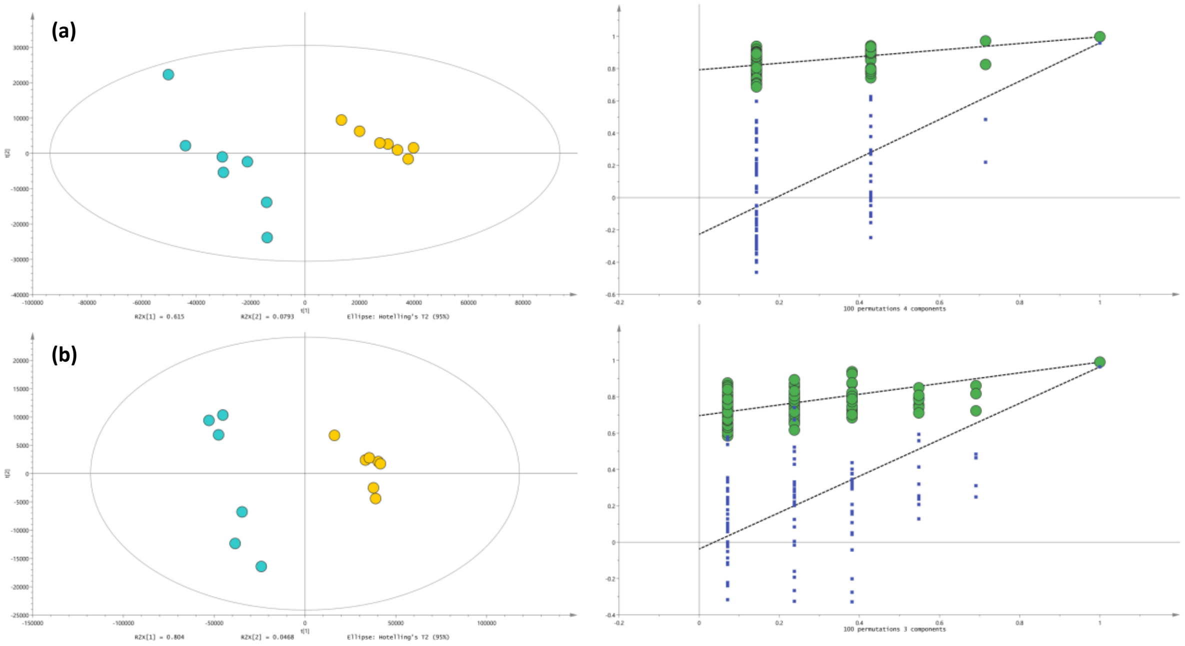

Supplementary Figure S7. Partial least square analysis – Discriminant analysis (PLS-DA) and permutation test (100 rearrangements) – Comparison of UHPLC-HRMS metabolic profiles from *A. officinalis* root (**a**) and shoot (**b**) samples associated with four different AMF species after 9 days of growth in the semi-hydroponic cultivation system (*R. intraradices* MUCL 49410: green dots; *R. clarus* MUCL 4623: red dots).

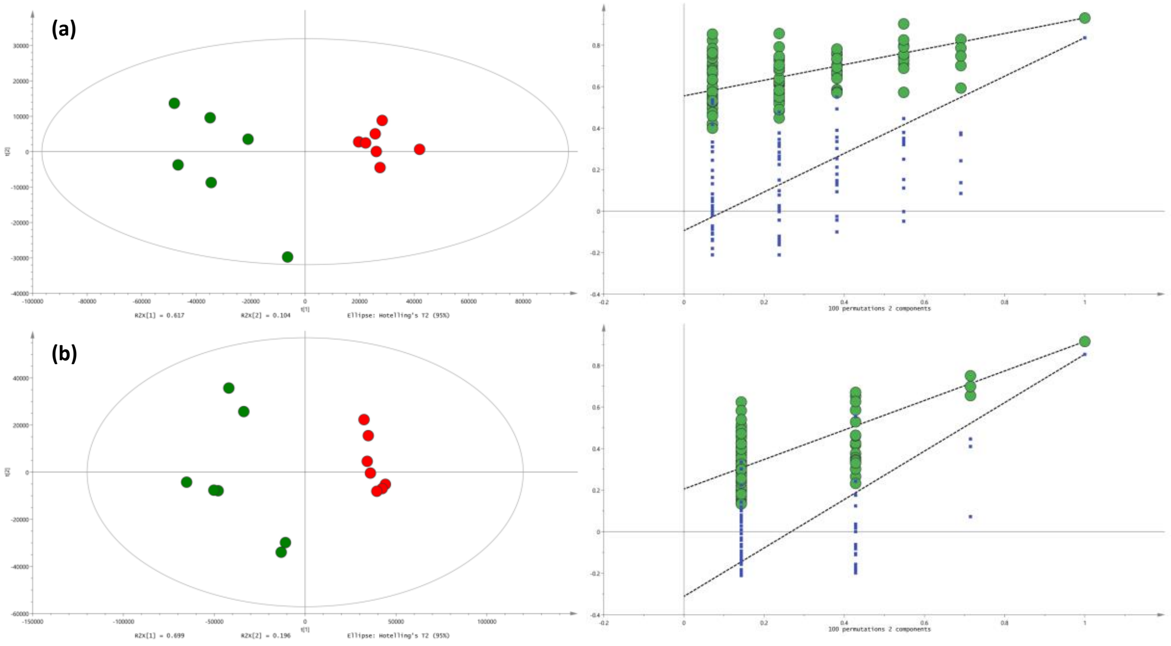

Supplementary Table S1. Evolution of AMF-root colonization and total fresh weight (TFW) of *A. officinalis* associated to one AMF species (*R. irregularis* MUCL 41833 –M^{irreg}, *R. intraradices* MUCL 49410 –M^{intra}, *R. clarus* MUCL 46238 –M^{clarus} and *R. aggregatus* MUCL 49408 –M^{aggreg}) before (T0) and after 9 days (T1) of growth in the S-H cultivation system.

		**AMF-root colonization (%)**		**Fresh weight (g)**
**AMF treatments**	**Harvest time**	**TC**	**AC**	**TFW**
MUCL 41833-M^{irr}	T0	76 ± 3	14 ± 3	5.74 ± 1.5
MUCL 49410-M^{intra}	T0	91 ± 3	22 ± 3	7.78 ± 1.5
MUCL 46238-M^{clarus}	T0	86 ± 3	18 ± 3	4.95 ± 1.5
MUCL 49408-M^{aggreg}	T0	85 ± 3	17 ± 3	7.73 ± 1.5
MUCL 41833-M^{irr}	T1	63 ± 3	6 ± 3	5.63 ± 1.5
MUCL 49410-M^{intra}	T1	70 ± 3	12 ± 3	8.66 ± 1.5
MUCL 46238-M^{clarus}	T1	61 ± 3	6 ± 3	5.31 ± 1.5
MUCL 49408-M^{aggreg}	T1	68 ± 3	10 ± 3	8.39 ± 1.5

The parameters measured are expressed as mean ± standard errors (SE) of 7 (T0) and 7 (T1) replicates per each AMF treatment.
